# Evaluating Traditional Chinese Medicine Interventions on Chronic Low Back Pain Using Goal Attainment Scaling

**DOI:** 10.1155/2020/8854927

**Published:** 2020-11-28

**Authors:** Xinyu Zhang, Jianping Ren, Chaojie Liu, Mengyan He, Lixian Ren, Zhen Lv

**Affiliations:** ^1^Department of Health Management, School of Medicine, Hangzhou Normal University, Hangzhou 311121, China; ^2^Department of Health Assessment, School of Health Management, Jinzhou Medical University, Jinzhou 121000, China; ^3^School of Psychology and Public Health, La Trobe University, Melbourne VIC 3086, Australia; ^4^Jiangcun Community Health Service Center, Xihu District, Hangzhou 310030, China

## Abstract

**Background:**

Extensive studies have been conducted to evaluate the pain relief effect of traditional Chinese medicine (TCM) interventions on patients with low back pain, including in China. However, there is a dearth in the literature documenting the diverse goals of TCM interventions, let alone the overall effect of such interventions. In this study, the goal attainment scaling (GAS) method was adopted to evaluate individualised TCM interventions on chronic low back pain.

**Methods:**

A pre-post intervention study was conducted on patients with chronic low back pain who received individualised TCM interventions in community health services. The study was undertaken in three community health centres in Hangzhou of China. A total of 165 eligible patients were invited, and 150 participated in the study, including 136 who completed both pre- and postintervention surveys. Each participant was asked to identify three to five intended goals from a pool of 26 outcome indicators and their corresponding expectations of these goals prior to the TCM interventions. Their conditions were rated against the selected indicators on a self-report five-point Likert scale before and after the TCM interventions, respectively. Gaps between the actual conditions and the expected goals were summed up for each participant and converted into a standardised GAS score, with a higher score indicating higher achievements, and 50 indicting patient expectations were met. Linear regression models were established to determine the factors associated with the pre-post GAS changes after adjustment for variations in other variables.

**Results:**

On average, an increase of 14.99 (SD = 9.81) in the GAS scores was achieved. This resulted in a mean GAS score of 48.33 (SD = 9.74) after the TCM interventions, falling slightly short (<2) of patient expectations. The multivariate linear regression models revealed that local residents, the retired, and those who perceived lower professional competency of their attending doctors had a smaller increase in the GAS scores after adjustment for variations in other variables.

**Conclusion:**

The individualised TCM interventions can help patients with low back pain to achieve their expected goals as measured by the GAS. Further studies are needed to better understand how patients set up their goals and the professional competency requirements to meet patient expectations.

## 1. Background

Chronic low back pain is a highly prevalent condition that can seriously jeopardise the way people function and their quality of life. A systematic review identified 28 studies reporting the prevalence of chronic low back pain [1]. Variations in the study samples and data definition and collection methods in these studies generated diverse results, with a prevalence estimation ranging from 2% to 25% in adult populations. Some researchers believe that the world-wide prevalence of low back pain is likely to be around 23% [1, 2]. Empirical studies [1] found that women are more likely to suffer from low back pain than men. For those aged between 30 and 60 years, the prevalence of chronic low back pain increases linearly. Many socioeconomic factors such as high workloads, low job control, and a lack of social support also contribute to the high prevalence of chronic low back pain. Chronic manifestation of pain is associated with a loss of productivity, emotional distress, and limitations in activities of daily living [2].

Common strategies for managing low back pain include self-management programs, physical exercise, primary care interventions using a biopsychosocial approach, and spinal manipulative therapy [3–5]. A medium level compared with a low level of physical activity was proved to be associated with a lower odds of low back pain (adjusted odds ratio = 0.93) [6]. There is moderate-quality evidence to support the effectiveness of self-management programs on reductions in pain intensity and disability [3]. The biopsychosocial interventions that focus explicitly on helping patients to understand pain, alleviate helpless thoughts, establish adequate goals, and develop coping strategies seem to be the most promising strategy [4]. They are more effective than education/counseling alone and are as effective as physical exercise interventions. A systematic review conducted by Rubinstein and colleagues found that spinal manipulative therapy can also have similar effects on chronic low back pain as other recommended therapies [5].

Traditional Chinese medicine (TCM) interventions have been adopted as an alternative approach to pain management in many healthcare settings [7, 8]. These usually include acupuncture, moxibustion, Chinese massage, cupping, and Chinese herbal medicine. In the US, acupuncture has been integrated into the comprehensive pain management plans for millions of older Americans [9]. The purpose of TCM interventions often goes beyond the scope of pain control. This is particularly true in Asian countries where TCM has been recognised by the mainstream health system. For example, moxibustion therapy was found to be effective not only in reducing low back pain but also in improving sleep quality and the functions of daily living in a South Korean study [10]. In China, TCM has been promoted by the government as a health promotion tool, providing people-centred care to maintain overall health and managing chronic conditions. TCM is considered as a part of the primary care system that is widely available, cheap, and convenient. TCM services are expected to offer a good patient experience and help alleviate cost pressures and exorbitant financial burdens on patients, families, and society [11, 12]. Indeed, the philosophy of TCM emphasises a whole-person view, which aligns well with the concept of people-centred care that concerns the health and wellbeing of patients from a variety of perspectives, not only physical, but also emotional, mental, spiritual, social, and financial [13]. TCM also advocates individualised care, treating each patient differently [14].

Extensive studies have been conducted to evaluate the pain relief effect of TCM interventions on patients with low back pain, including in China [15]. However, there is a dearth in the literature documenting the diverse goals of TCM interventions, let alone the overall effect of such interventions. This study fills the gap in the literature, with the aim of evaluating a comprehensive TCM program that applied individualised TCM interventions involving various TCM modalities and treatment goals. The evaluated TCM program also incorporated modern information technologies into TCM consultations and patient education.

The lack of a standardised intervention protocol (and predefined intervention goals) for TCM treatment presents a great challenge to the design of this evaluation study. The literature review identified six instruments that were developed for measuring individualised health outcomes, including the goal attainment scaling (GAS) [16–21]. We chose the GAS instrument for the purpose of this study because it is a general instrument which is not tailored to any specific disease conditions and it allows care providers to negotiate treatment goals with their patients which reflect the actual care process. Several studies [22, 23] have called for applications of individualised outcome evaluations in complementary and alternative medicine. The misalignment between clinical outcome measures (e.g., pain) and patient-derived goals for the management of chronic low back pain has become a particular concern [24]. In China, community health services aim at improving the quality of life of patients. The GAS method provides a useful tool to evaluate the patient-centered care outcomes in line with the mission of community health services. The principles of GAS align particularly well with the individualised whole-of-person approach in TCM interventions as an essential part of community health services in China. The GAS instrument can quantify the achievements of individualised interventions, which not only enables the comparability of intervention outcomes but also provides details about the gaps in services.

## 2. Methods

A pre-post intervention study was conducted on 136 patients with chronic low back pain. The study was undertaken in three urban community health centres (CHCs) in Hangzhou, one of the most developed municipalities in China with over 9.8 million permanent residents. Hangzhou is divided into 13 local jurisdictions. Over 77% of its population resides in the 10 urban districts. There are 129 urban CHCs in Hangzhou. Of these centres, 100 have a dedicated unit providing TCM services. The revenue generated from TCM services can contribute to as high as 31% of the total revenue of a CHC [25].

Ethics approval for the study protocol was obtained from Hangzhou Normal University (reference number 20190070).

### 2.1. Sampling

The variation (*σ*) of the GAS score was estimated to be 5.89 according to the preintervention survey. To detect an effect size (*δ*) of 2, a minimal sample size of 113 is required with a statistical power (*β*) of 0.80 at the statistical significance level (*α*) of 0.05 [26]:

(1) $$\begin{array}{c} N={\left[\frac{\left(Z_{\alpha /2}+Z_{\beta }\right)\sigma_{d}}{\delta }\right]}^{2}={\left[\frac{\left(1.96+1.645\right){}^{*}5.89}{2}\right]}^{2}=113. \end{array}$$

A stratified purposive sampling strategy was adopted to select participants. Three urban districts with different levels of socioeconomic status were identified first: Xihu representing the most developed districts, Gongshu representing the least developed districts, and Shangcheng sitting between the two. One average-sized CHC from each of the three districts was then selected. Eligible CHCs were those with a TCM unit. Study participants were recruited from these units over the period from 1 to 19 July 2019. The patients who sought TCM services for chronic low back pain were invited by the TCM doctors to participate in this study based on the following inclusion criteria: (1) being 40 years of age or older; (2) agreeing to register in the TCM care management program in line with the diagnostic and therapeutic efficacy standards for chronic low back pain formulated by the State Administration of TCM (2017); (3) not having received any TCM services over the past four weeks. Participation was voluntary. The eligibility of the participants was assessed by the TCM doctors. They were deemed to be safe to receive TCM-only interventions. Over the study period, the participants did not receive any other treatments such as analgesic drug and physical therapy apart from the TCM interventions. Those with severe cognitive impairment, aphasia, and heart, liver, kidney, psychiatric disorders, and other serious conditions that required allopathic medicine interventions were excluded from the study. Those who were unable to understand and provide informed consent were also excluded from the study.

### 2.2. Intervention

The interventions were conducted by a team of CHC workers, each comprising a TCM doctor and three nurses. A comprehensive package of TCM interventions was applied to the study participants over a four-week period. The measures included (1) one-to-one TCM consultations to identify the “constitution” (Tizhi in Chinese which refers to the innate condition influenced by the environment that “integrates the morphological structure and physiological function with psychological state”) of the patient [27] for the purpose of developing an individualised care plan; (2) provision of acupuncture, moxibustion, and other relevant TCM services as applicable to each individual patient once every other day; (3) communication and education through the WeChat groups on Wednesdays and Saturdays (including eight sessions of TCM knowledge training in the evening of Saturdays covering diet, bed, posture, housework, bending and lifting, foot care, and physical exercise); and (4) telephone follow-up consultations designed for those who did not have access to smartphones and the WeChat groups. All of the participants received the above interventions; however, the services plan (such as the applicable TCM modalities and points of acupuncture) for each patient depended on the comprehensive pattern (constitution) diagnosis. Despite the individualised plans, the frequency and length of each TCM intervention session were kept consistent: around 30 minutes. In total, 61 (40.7%) patients joined the WeChat groups, compared with 89 (59.3%) who received telephone follow-up services. Each WeChat group had a patient champion who collected additional questions and liaised with the TCM doctors for corresponding answers.

### 2.3. Outcome Measurements

The Goal Attainment Scaling (GAS) was adopted to evaluate the outcomes of the TCM interventions. The GAS method was developed by Kirusek and Sherman in the US [16], which allows the service recipients (patients) to identify their expected goals in conjunction with their service providers. It is not just a tool, but a process or method that can meet the needs of every new situation albeit at the cost of difficulties in ascertaining psychometric properties [28]. In this study, each patient was asked to choose three to five preferred targets. To enable a comparison of the outcomes across patients, all targets were assessed on the same five-point Likert scale, ranging from “−2, the worst possible outcome,” to “2, the best possible outcome.” The larger the score, the better the patient's condition. A score of 0 indicates a level that just meets the agreed successful goal. A weighted GAS score was calculated by summing up the gaps between the current conditions and the expected outcomes of each patient on her/his identified targets [29]:

(2) $$\begin{array}{c} \text{GAS}=50+\frac{10\sum \left(W_{i}X_{i}\right)}{\sqrt{0.7\sum W_{i}^{2}+0.3{\left(\sum W_{i}\right)}^{2}}}, \end{array}$$

where *X*_i_ represents the gap score of the *i*-th target and *W*_*i*_ is the weight assigned to the *i*-th target. In this study, the weight for each target considered its importance (*W*_imp_) rated by the patient on a three-point scale ranging from 1 “least important” to 3 “most important” and its difficulty (*W*_dif_) rated by the TCM doctor on a three-point scale ranging from 1 “least difficult” to 3 “most difficult”: *W*_*i*_ = *W*_imp_ × *W*_dif_. This combined the gap scores of various targets into a single GAS score, measuring the extent to which the individual goals were achieved. A GAS score of 50 represents the predefined goal of successful interventions.

To simplify the process and make it easier for the patients to identify relevant targets, a pool of potential targets was developed by the research team based on the functionalities embedded in the International Classification of Function, Disability, and Health (ICF) [30]. Through interviews with six TCM doctors and 14 patients with chronic low back pain and consultations with 18 experts on the relevance, importance, and applicability of the targets, a total of 26 target indicators were developed covering seven aspects of health functioning, activities of daily living, and social participation (Table 1).

### 2.4. Data Collection

Two rounds of questionnaire surveys were undertaken through face-to-face interviews. Three interviewers were recruited from the Hangzhou Normal University and trained to follow the standard protocol. The interviews were conducted in the waiting room or the acupuncture room in the participating CHCs. Each completed questionnaire was checked by another investigator to ensure completeness and that it was free of logical errors.

The first round of (preintervention) questionnaire surveys was conducted from 1 to 19 July 2019, tapping into the sociodemographic characteristics (gender, ethnicity, age, residency, education, marital status, household income, employment, occupation, and medical insurance) and health conditions (chronic disease, seriousness and duration of low back pain, and self-rated health) of the respondents. These variables were selected in line with the social determinants of health theory. Previous studies show that these variables have a significant impact on health and health care outcomes [31, 32]. Residency was defined in line with China's household registration (Hukou) system: those without a Hangzhou Hukou are not eligible for the welfare (e.g., housing, education, employment, and insurance) entitlements enjoyed by the local (Hangzhou) Hukou holders. China has established almost universal health insurance coverage, but with many funds based on the Hukou system [32]. Overall, urban employees enjoy a higher level of social health insurance entitlements compared with their rural and other urban resident counterparts. In this study, the level of pain was assessed using a visual analogue scale (VAS) ranging from 0 to 10. A single item was designed to assess self-rated health on a three-point Likert scale. The questionnaire also asked respondents to rate their perceptions of the professional competency of attending TCM doctors in comparison with the average of the professional body.

During the preintervention survey, the study participants were asked to select three to five targets from the indicator pool that they considered most relevant and important, to rate their status and to indicate their expected goal on the scale of each target indicator.

The second round of (postintervention) surveys was conducted from 5 to 23 August 2019 after the conclusion of the four-week TCM interventions. The identity of the respondents was matched with those recorded in the first round of survey. They were again asked to rate their status on the scale of each target indicator.

A total of 165 eligible patients were approached in the preintervention survey, and 150 (90.9%) returned a valid questionnaire. Of the 150 participants, 136 (90.7%) completed the postintervention survey: 12 (8.0%) were lost in follow-ups, and 2 (1.3%) rejected the follow-up invitations.

### 2.5. Data Analysis

Data were entered into EpiData and analysed using SPSS v21.0. A *p* value of <0.05 was considered statistically significant.

The characteristics of the study participants were described through frequency distributions. Mean values and standard deviations (SD) of the GAS scores were presented for the participants before and after the TCM interventions. The pre-post intervention differences in the GAS scores were tested using paired *t* tests. Changes in the GAS scores were compared between the participants with various characteristics through student *t*-tests or analysis of variance (ANOVA). A multivariate linear regression model was established to determine the factors associated with the pre-post changes in the GAS scores after adjustment for variations in other variables. An enter approach was adopted in the regression modelling.

## 3. Results

### 3.1. Characteristics of Respondents

The study participants were predominantly female (63.2%). Most were married (94.9%) at the time and had a local household registration (78.7%) in Hangzhou. More than half of the respondents were older than 60 years (58.9%), had a monthly household income of 3001–6000 yuan per capita (54.4%), suffered from low back pain for less than five years (58.1%), and reported no coexisting chronic diseases (52.0%). The majority were retired (77.2%) and covered by the basic medical insurance for urban employees (64.0%). About 57.4% of the respondents reported good general health and 50.1% rated a level of pain ranging from 4 to 7 out of a possible 10 (Table 2).

### 3.2. Pre-Post Intervention Differences in GAS Scores

The mean GAS values increased from 33.35 (SD = 5.89) before the TCM interventions to 48.33 (SD = 9.74) after the TCM interventions (*t* = −17.817, *p* < 0.01). The achievement was slightly below the successful level of 50. About 36.0% of participants reported an achievement meeting or exceeding the set goals after the TCM interventions.

Despite a universal improvement in the GAS scores for all the study participants with various characteristics, the local patients showed a lower level of increase in the GAS values than their nonlocal counterparts. Lower levels of increase in the GAS values were found in those who were retired and had more chronic diseases. A higher level of increase in the GAS values was also associated with higher perceptions of the professional competency of the attending doctors (Table 2).

Pain relief was the most common goal (100%) selected by the study participants, followed by walking function (50.7%), standing (40.0%), and basic body posture change (38.2%). For the goals selected by at least 20 participants, more than 60% of respondents reported achieving the goals (Table 3).

The multivariate linear regression model confirmed that nonlocal residency (*β* = 0.172, *p*=0.034) and higher perceptions of the professional competency of attending doctors (*β* = 0.380, *p* < 0.001) were associated with higher postintervention improvements in the GAS scores, whereas retirement (*β* = −0.160, *p*=0.048) was associated with lower postintervention improvements in the GAS scores. The effect of coexisting chronic diseases became statistically insignificant after adjustment for variations in other variables (Table 4). Further analyses on the postintervention GAS scores and associated factors indicated that the pre-post changes in the GAS values were mainly attributable to the variations in the postintervention GAS scores.

## 4. Discussion

This study shows that the individualised TCM interventions in community health services can improve patient-reported health outcomes as measured by the GAS. The TCM intervention program evaluated in this study involves multiple TCM modalities tailored to the specific needs of each patient with chronic low back pain. The target outcome indicators chosen by each patient vary in nature and numbers. The GAS results demonstrated an increase of 14.99 in the GAS scores (SD = 9.81) on average for each participant over a one-month period. Overall, the TCM interventions are successful, albeit falling slightly short of patient expectations. About 36.0% of patients successfully achieved their set goals. This result is consistent with findings of studies conducted elsewhere [33, 34]. It is important to note that the GAS targets were set up by the patients. Both high patient expectations and low performance of the TCM services might contribute to the results. Indeed, patient perceptions of the professional competency of the attending doctor were shown to be an independent predictor of the postintervention improvement in the GAS scores after adjustment for variations of other variables.

In this study, nonlocal patients reported a higher level of postintervention achievements than their local counterparts although their perceptions of the professional competency of the attending doctors remained consistent. It is not clear whether this is a result of the lower expectations of the nonlocal patients. Although we do not have direct evidence to demonstrate that nonlocal residents hold a lower level of expectations of the services they received, previous studies showed that nonlocal residents tend to seek cheaper health care services due to financial barriers and a lack of local support and welfare entitlements [35, 36]. This may have inevitably lowered their expectations.

Surprisingly, socioeconomic factors were not found to be associated with the levels of postintervention achievements, although the retirees had a lower level of improvements. Great socioeconomic disparities exist in China. Wang et al. [37] found in a study that income is the single most important predictor of patient access to outpatient care in both the least developed province (Gansu) and the most developed province (Zhejiang) in China. Despite great progress in the universal coverage of social health insurance, out-of-pocket medical spending has continued to be a serious financial burden on consumers, particularly for those who are covered by less generous insurance programs such as those for urban and rural residents [38]. However, neither household income nor health insurance coverage was found to be associated with the GAS scores in this study.

We call for further studies into applications of the GAS instrument to evaluate patient-reported health care outcomes. This instrument is particularly useful in a health care environment that encourages highly individualised patient-centred care such as TCM. Several lessons can be learnt from this study. Firstly, a predefined pool of target indicators can be developed to make it easier for care providers to negotiate with their patients in setting up goals. A consensus on the target pool needs to be reached between care providers and patients. Secondly, there is a need to test whether a general pool of targets, such as the one developed in this study based on the ICF, can be used across several disease conditions, possibly through the assignment of different weights on the target indicators in line with the expectations of different patients. Thirdly, the GAS methods can be understood by patients with limited education. However, it will be a great challenge to ask patients to set up goals without a reference pool of targets, especially in a culture where the authority of doctors is highly respected.

## 5. Limitations

This study adopted a pre-post intervention design without a control group due to ethical concerns as TCM interventions on chronic low back pain have been promoted by the health authorities in China. As a result, the Hawthorn effect cannot be completely ruled out. The study was conducted in Hangzhou, one of the cities with the highest concentration of quality TCM resources in community health services [39, 40]. Attempts to extrapolate the findings to other regions need to be cautious.

## 6. Conclusions

TCM services provide individualised interventions on patients with chronic low back pain. This study shows that the target goals set up by the patients can be improved through individualised TCM interventions, albeit at a level that falls slightly short of expectations. Further improvements can be made through strengthening the professional competence of TCM doctors, which is also advocated by other researchers [41].

## Figures and Tables

**Table 1:** Target pool for TCM interventions on low back pain.

Domain	Function	Target
1. Health functioning	1.1 Mental function	1.1.1 Sleeping
		1.1.2 Emotion
	1.2 Pain and physical function	1.2.1 Sense of pain
		1.2.2 Exercise tolerance
		1.2.3 Joint function
2. Daily activity	2.1 Daily routine	2.1.1 Performance of multiple tasks
		2.1.2 Performance of daily routine
	2.2 Daily activities	2.2.1 Basic body posture change
		2.2.2 Sitting
		2.2.3 Standing
		2.2.4 Lifting and carrying objects
		2.2.5 Walking
		2.2.6 Moving around
		2.2.7 Using transportation
		2.2.8 Driving
		2.2.9 Bathing
		2.2.10 Dressing
		2.2.11 Self-care
3. Participation	2.3 Family function	2.3.1 Shopping
		2.3.2 Housework
		2.3.3 Family relationship
	2.4 Social function	2.4.1 Remunerative employment
		2.4.2 Community activities
		2.4.3 Leisure activities
	3.1 Social support	3.1.1 Family support for material or emotional needs
		3.1.2 Physical or emotional support from professionals

**Table 2:** Changes in goal attainment scores by sociodemographic characteristics of study participants.

Characteristics	*n*	(%)	GAS score (Mean ± SD)			Pre-post GAS difference	*F*
			Preintervention	Postintervention	Paired *t*		
Gender							0.013
Male	50	(36.8)	32.03 ± 6.93	46.89 ± 10.29	−10.485^*∗∗*^	14.86 ± 10.02	
Female	86	(63.2)	34.11 ± 5.08	49.17 ± 9.36	−14.335^*∗∗*^	15.06 ± 9.74	
Age (years)							2.174
40–49	24	(17.6)	31.95 ± 6.30	51.06 ± 8.49	−10.681^*∗∗*^	19.11 ± 8.77	
50–59	32	(23.5)	32.42 ± 6.14	47.96 ± 9.31	−8.576^*∗∗*^	15.53 ± 10.25	
60–69	50	(36.8)	33.36 ± 5.91	47.31 ± 11.37	−9.744^*∗∗*^	13.95 ± 10.13	
≥70	30	(22.1)	35.43 ± 4.86	48.26 ± 8.08	−7.831^*∗∗*^	12.82 ± 8.97	
Residency							4.125^*∗*^
Local	107	(78.7)	33.37 ± 5.93	47.48 ± 9.81	−15.547^*∗∗*^	14.11 ± 9.39	
Nonlocal	29	(21.3)	33.26 ± 5.85	51.49 ± 8.95	−9.092^*∗∗*^	18.23 ± 10.80	
Education							1.527
≤primary school	46	(33.8)	33.68 ± 5.68	47.23 ± 10.28	−10.157^*∗∗*^	13.54 ± 9.04	
Middle school	40	(29.4)	33.79 ± 4.73	47.87 ± 9.60	−8.258^*∗∗*^	14.08 ± 10.78	
High school	25	(18.4)	33.82 ± 6.32	49.47 ± 8.08	−7.849^*∗∗*^	15.65 ± 9.97	
University	25	(18.4)	31.55 ± 7.36	49.98 ± 10.65	−10.211^*∗∗*^	18.43 ± 9.02	
Marital status							1.217
Not Married	7	(5.1)	30.47 ± 6.45	49.44 ± 11.26	−6.814^*∗∗*^	18.97 ± 7.36	
Married	129	(94.9)	33.5 ± 5.84	48.27 ± 9.70	−16.943^*∗∗*^	14.77 ± 9.90	
Monthly household income per capita (¥)							0.696
≤3000	41	(30.1)	33.05 ± 5.60	46.91 ± 11.48	−9.138^*∗∗*^	13.86 ± 9.71	
3001–6000	74	(54.4)	33.21 ± 6.13	48.26 ± 9.00	−12.317^*∗∗*^	15.05 ± 10.51	
>6000	21	(15.4)	34.41 ± 5.72	51.38 ± 8.22	−10.899^*∗∗*^	16.96 ± 7.13	
Employment							6.560^*∗*^
Employed	31	(22.8)	31.55 ± 6.76	50.43 ± 7.29	−12.945^*∗∗*^	18.87 ± 8.12	
Retired	105	(77.2)	33.88 ± 5.53	47.72 ± 10.30	−14.176^*∗∗*^	13.84 ± 10.00	
Job							1.233
Manager	30	(22.1)	32.87 ± 6.98	48.92 ± 10.23	−8.639^*∗∗*^	16.05 ± 10.18	
Office worker	25	(18.4)	33.33 ± 6.78	45.03 ± 10.83	−6.400^*∗∗*^	11.70 ± 9.14	
Laborer	54	(39.7)	33.69 ± 5.10	48.95 ± 8.65	−11.769^*∗∗*^	15.27 ± 9.53	
Self-employed	27	(19.9)	33.21 ± 5.47	49.50 ± 10.10	−8.138^*∗∗*^	16.29 ± 10.40	
Health insurance							0.617
Urban employee	87	(64.0)	32.83 ± 6.22	48.07 ± 9.98	−14.091^*∗∗*^	15.24 ± 10.09	
Urban residents	33	(24.3)	33.63 ± 4.88	49.18 ± 9.47	−9.092^*∗∗*^	15.55 ± 9.83	
Rural and others	16	(11.8)	35.55 ± 5.70	48.00 ± 9.45	−6.006^*∗∗*^	12.45 ± 8.29	
Duration of chronic low back pain (years)							0.354
≤5	79	(58.1)	32.85 ± 6.22	47.75 ± 9.71	−13.003^*∗∗*^	14.90 ± 10.19	
6–10	27	(19.9)	33.76 ± 5.15	50.01 ± 10.62	−8.236^*∗∗*^	16.25 ± 10.25	
>10	30	(22.1)	34.28 ± 5.64	48.35 ± 9.13	−9.067^*∗∗*^	14.07 ± 8.50	
Coexisting chronic diseases							3.605^*∗*^
0	68	(50.0)	32.60 ± 6.36	49.66 ± 9.34	−13.888^*∗∗*^	17.06 ± 10.13	
1	50	(36.8)	34.35 ± 5.24	47.93 ± 9.47	−10.355^*∗∗*^	13.58 ± 9.27	
≥2	18	(13.2)	33.38 ± 5.64	44.45 ± 11.32	−5.507^*∗∗*^	11.07 ± 8.53	
Perceived health							0.618
Poor	36	(26.5)	32.14 ± 6.60	45.57 ± 11.09	−7.900^*∗∗*^	13.42 ± 10.20	
Fair	65	(47.8)	34.24 ± 5.58	49.77 ± 9.31	−11.730^*∗∗*^	15.53 ± 10.67	
Good	35	(25.7)	32.92 ± 5.58	48.51 ± 8.66	−12.201^*∗∗*^	15.59 ± 7.56	
Distance to nearest community health centre (minutes)							0.043
≤15	67	(49.3)	32.54 ± 6.25	47.43 ± 10.11	−12.526^*∗∗*^	14.89 ± 9.73	
16–30	28	(20.6)	33.11 ± 6.40	48.58 ± 9.26	−9.476^*∗∗*^	15.47 ± 8.64	
>30	41	(30.1)	34.84 ± 4.64	49.65 ± 9.52	−8.733^*∗∗*^	14.81 ± 10.86	
Perceived professional competency of attending doctor							21.283^*∗∗∗∗*^
≤average	58	(42.6)	33.84 ± 6.41	44.63 ± 9.00	−8.536^*∗∗*^	10.79 ± 9.63	
>average	78	(57.4)	32.98 ± 5.49	51.09 ± 9.40	−18.226^*∗∗*^	18.11 ± 8.77	
Pain level							1.125
≤4	57	(41.9)	32.90 ± 5.79	49.25 ± 8.93	−13.619^*∗∗*^	16.36 ± 9.07	
4.1–7	69	(50.7)	33.85 ± 6.12	47.61 ± 10.62	−10.563^*∗∗*^	13.76 ± 10.82	
>7	10	(7.4)	32.46 ± 4.96	48.11 ± 8.09	−10.216^*∗∗*^	15.66 ± 4.85	

Note:^*∗∗*^*p* < 0.01, ^*∗*^*p* < 0.05.

**Table 3:** The improvement in goal achievement in GAS.

Target	N	Preintervention Mean±SD	Postintervention Mean±SD	Pre-post Difference Mean±SD	Postintervention (%)with a score ≥ 0 (goal achieved)
1.1.1 Sleeping	51	−1.18 ± 0.39	−0.12 ± 0.91^*∗*^	1.06 ± 0.90	69
1.1.2 Emotion	18	−1.00 ± 0.00	0.17 ± 0.79^*∗*^	1.17 ± 0.79	72
1.2.1 Sense of pain	136	−1.14 ± 0.41	−0.15 ± 0.79^*∗*^	0.99 ± 0.77	74
1.2.2 Exercise tolerance	6	−1.00 ± 0.00	0.50 ± 1.38^*∗*^	1.50 ± 1.38	67
1.2.3 Joint function	39	−1.08 ± 0.27	0.03 ± 0.63^*∗*^	1.10 ± 0.68	85
2.1.1 Performance of multiple tasks	3	−1.00 ± 0.00	−0.67 ± 0.58	0.33 ± 0.58	33
2.1.2 Performance of daily routine	6	−1.17 ± 0.41	0.00 ± 0.63^*∗*^	1.17 ± 0.75	83
2.2.1 Basic body posture change	52	−1.12 ± 0.32	0.06 ± 0.85^*∗*^	1.17 ± 0.81	78
2.2.2 Sitting	48	−1.33 ± 0.48	−0.04 ± 1.05^*∗*^	1.29 ± 0.99	71
2.2.3 Standing	53	−1.38 ± 0.49	−0.38 ± 0.86^*∗*^	1.00 ± 0.76	74
2.2.4 Lifting and carrying objects	32	−1.22 ± 0.42	−0.09 ± 0.78^*∗*^	1.13 ± 0.91	72
2.2.5 Walking	69	−1.38 ± 0.49	−0.23 ± 0.94^*∗*^	1.14 ± 0.93	71
2.2.8 Driving	5	−1.20 ± 0.45	0.80 ± 1.30^*∗*^	2.00 ± 1.58	80
2.2.10 Dressing	7	−1.14 ± 0.69	−0.71 ± 0.76	0.43 ± 0.79	57
2.2.11 Self-care	6	−1.33 ± 0.52	−0.33 ± 1.21	1.00 ± 1.10	67
2.3.1 Shopping	8	−1.25 ± 0.46	−0.50 ± 1.20	0.75 ± 1.17	63
2.3.2 Housework	29	−1.17 ± 0.47	−0.28 ± 0.65^*∗*^	0.90 ± 0.72	69
2.4.1 Remunerative employment	10	−1.30 ± 0.48	−0.10 ± 1.29^*∗*^	1.20 ± 1.14	70
2.4.3 Leisure activities	14	−1.21 ± 0.43	−0.43 ± 0.85^*∗*^	0.79 ± 0.70	36
3.1.2 Physical or emotional support from professionals	9	−1.00 ± 0.00	0.22 ± 0.44^*∗*^	1.22 ± 0.44	100

Note:^*∗*^*p* < 0.05 in comparison with preintervention scores.

**Table 4:** Factors associated with pre-post intervention changes in GAS score results of the multivariate linear regression model.

Predictor	*β*	*t*	*p*
Constant	—	6.247	0.000
Gender (ref. = male)			
Female	0.082	1.024	0.308
Age (ref. = 40–49 years)			
50–59	−0.011	−0.146	0.884
60–69	0.057	0.667	0.506
≥70	−0.078	−0.956	0.341
Residency (ref. = local)			
Nonlocal	0.172	2.147	0.034
Education (ref. = primary school and below)			
Middle school	−0.032	−0.403	0.688
High school	−0.013	−0.157	0.875
University	0.060	0.726	0.469
Marital status (ref. = not married)			
Married	−0.110	−1.404	0.163
Monthly household income per capita (ref. = ≤ ¥3000)			
3001–6000	0.013	0.161	0.872
≥6000	−0.060	−0.721	0.472
Employment (ref. = employed)			
Retired	−0.160	−2.000	0.048
Job (ref. = manager)			
Office worker	−0.076	−0.958	0.340
Laborer	0.035	0.439	0.662
Self-employed	0.029	0.362	0.718
Health insurance (ref. = urban employee)			
Urban residents	0.090	1.153	0.251
Rural and others	−0.113	−1.440	0.152
Duration of chronic low back pain (ref. = ≤5 years)			
5–10	0.067	0.844	0.400
>10	−0.017	−0.223	0.824
Number of other chronic diseases (ref. = 0)			
1	−0.045	−0.561	0.576
≥2	−0.096	−1.203	0.231
Perceived health (ref. = poor)			
Fair	0.024	0.300	0.765
Good	0.016	0.204	0.839
Distance to nearest community health centre (ref. = ≤15 minutes)			
15∼30	0.016	0.205	0.838
>30	−0.058	−0.740	0.461
Perceived professional competency of attending doctors (ref. = “≤average”)			
>Average	0.380	4.860	0.000
Pain level (ref. = 0–4)			
4.1–7	−0.093	−1.186	0.238
>7	0.019	0.247	0.805

Note: *F* = 11.339, *p* < 0.01, *R*^*2*^ = 0.187.

## Data Availability

The data used to support the findings of this study are available from the corresponding author.

## Abbreviations

GAS: Goal Attainment Scaling
TCM: Traditional Chinese medicine
CHCs: Community Health Centres
ICF: The International Classification of Function, Disability, and Health
VAS: Visual analogue scale.

## Supplementary Materials

Supplementary Materials Table S1: example of GAS target indicators identified by a patient. Table S2: GAS target indicator scores assigned by the patient. Table S3: gaps with expectations in target indicators and GAS scores.
