# Supplementary material for: Evaluating Traditional Chinese Medicine Interventions on Chronic Low Back Pain Using Goal Attainment Scaling
Source: Evid Based Complement Alternat Med. 2020 Nov 28;2020:8854927. doi: 10.1155/2020/8854927 (PMC7735859; doi:10.1155/2020/8854927)
Supplement: Supplementary Materials — Table S1: example of GAS target indicators identified by a patient. Table S2: GAS target indicator scores assigned by the patient. Table S3: gaps with expectations in target indicators and GAS scores. [file 8854927.f1.doc]

**Supplement File**

**Supplement File description:**

**Table S1: Example of GAS target indicators identified by a patient**

**Table S2: GAS target indicator scores assigned by the patient**

**Table S3: Gaps with expectations in target indicators and GAS scores**

**Example of a case using the GAS method assessing TCM intervention outcomes**

A patient with low back pain identified five target indicators (Table S1) from the predefined pool of 26 indicators, which include detailed descriptions of the five-point Likert scale for each target indicator (Table 1).

**Table S1: Example of GAS target indicators identified by a patient**

| **Target indicator** | **Score** | | | | |
| --- | --- | --- | --- | --- | --- |
|  | **-2** | **-1** | **0** | **+1** | **+2** |
| **Sense of pain** | Serious | Medium | Slight | Occasionally | Normal |
| **Joint function** | Obviously restricted | Obvious when amplitude is large | Slightly restricted | Occasionally restricted | Normal |
| **Basic body posture change** | Serious difficulty | Medium difficulty | Slight difficulty | Occasionally difficulty | Normal |
| **Standing** | Serious difficulty | Medium difficulty | Slight difficulty | Occasionally difficulty | Normal |
| **Housework** | Serious difficulty | Medium difficulty | Slight difficulty | Occasionally difficulty | Normal |

The patient had high expectations on two indicators (joint function and housework) and set up a score of “1” as the expected outcome for the four-week TCM intervention, compared with “-1” for “standing” and “0” for “pain and posture change” (Table S2).

**Table S2: GAS target indicator scores assigned by the patient**

| **Target indicators** | **Pre-intervention** | **Expected** | **Post-intervention** |
| --- | --- | --- | --- |
| **Sense of pain** | -1 | 0 | 0 |
| **Joint function** | -1 | 1 | 0 |
| **Basic body posture change** | -1 | 0 | 0 |
| **Standing** | -2 | -1 | -1 |
| **Housework** | -1 | 1 | 0 |

The gap between the actual condition and the expected goal was calculated for each target indicator pre- and post-intervention, respectively. Weighted GAS scores were then calculated based on the importance rated by the patient and the difficulty rated by the TCM doctor (Table S3).

**Table S3: Gaps with expectations in target indicators and GAS scores**

| **Target indicator** | **Gaps with expectations in target indicator** | | **Patient rated Importance (W_imp_)** | **Doctor rated Difficulty (W_dif_)** | **Weight**  **(W_imp_×W_dif_)** |
| --- | --- | --- | --- | --- | --- |
|  | **Preintervention** | **Post-intervention** |  |  |  |
| **Sense of pain** | -1 | 0 | 2 | 2 | 4 |
| **Joint function** | -2 | -1 | 1 | 2 | 2 |
| **Basic body posture change** | -1 | 0 | 1 | 2 | 2 |
| **Standing** | -1 | 0 | 1 | 2 | 2 |
| **Housework** | -2 | -1 | 2 | 1 | 2 |
| **Weighted GAS scores** | 30.25 | 45.06 |  |  |  |
